# Martensitic Transformation and Strengthening Mechanism in a 304 Stainless Steel Subjected to Wire Drawing

**DOI:** 10.3390/ma19112412

**Published:** 2026-06-05

**Authors:** Yongjie Yu, Wujing Fu, Feng Dai, Rengeng Li, Qingquan Lai

**Affiliations:** Key Laboratory for Light-Weight Materials, Nanjing Tech University, Nanjing 211816, China; 15655746032@163.com (Y.Y.); fuwujing@mat-jitri.cn (W.F.); fengdai621@163.com (F.D.)

**Keywords:** stainless steel, wire drawing, austenite, martensitic transformation, strengthening

## Abstract

Wire drawing is a key processing method for producing ultrahigh-strength stainless steel wires. In metastable austenitic steels, the strain-induced martensitic transformation is known to govern strain hardening. However, the transformation mechanism and kinetics behavior under wire drawing remain unclear due to the distinct deformation conditions compared to those of conventional loading modes. In this work, the microstructural evolution, transformation kinetics and strengthening behavior of the 304 stainless steel during cold wire drawing are systematically analyzed. The results show that the transformation is dominated by the austenite → twin→ α′-martensite pathway, with the ε-martensite effectively suppressed. The martensite fraction follows a sigmoidal evolution with the equivalent drawing strain and could be well described by the Olson–Cohen model. The yield strength is increased from 320 MPa to 2 GPa and exhibits a linear relationship with the martensite fraction, indicating a dominant composite strengthening mechanism. These findings clarify the deformation-mode-dependent transformation mechanism and its role in governing mechanical properties during wire drawing.

## 1. Introduction

Wire drawing is a widely used method for producing ultrahigh-strength wire products for various engineering applications. The process of wire drawing involves a gradual reduction in the cross-sectional diameter via dozens of die passes, finally achieving a very large accumulated deformation. It is a typical severe plastic deformation technique that is ready for application [1,2]. 304-type stainless steel is an important material for wire drawing, mainly due to its good drawability. But the significant strain hardening during wire drawing induces issues such as die wear and premature wire breakage, etc. [3]. Therefore, an in-depth understanding and proper control of the microstructure and mechanical properties during wire drawing are essential for process optimization [3].

The rapid strain hardening of metastable stainless steels during plastic processing is mainly due to the occurrence of strain-induced martensitic transformation (SIMT). An excellent review on this topic can be found in Refs. [4,5,6]. For the 304-type austenitic stainless steels, a relatively low stacking fault energy is involved (13–20 mJ/m^2^) [7,8]. During the plastic deformation, planar slip of dislocations is dominant in the austenite phase, which is followed by the formation of shear bands that consists of deformation twins and ε-martensite plates [9,10]. It is generally considered that the intersection of shear bands facilitates the nucleation of α′-martensite, and the progress of α′-martensitic transformation is presumably controlled by the continuous formation of shear band intersections and thus the α′-martensite nucleation [11,12,13,14]. Based on this physical scenario, the Olson–Cohen model and its modifications have been developed to describe the kinetics of strain-induced α′-martensitic transformation and to model the deformation process of metastable steels [15,16,17].

The previous investigations on SIMT in metastable austenitic stainless steels have focused mainly on the deformation modes of uniaxial tension and rolling. Several earlier studied on SIMT in the context of wire drawing have demonstrated the complexity associated with this particular deformation mode [18,19,20,21,22,23]. For example, the die pressure counteracts the volume expansion of the austenite-to-martensite structure change, and tends to suppress the transformation [24,25,26]. In addition, the high strain rate of the wire drawing process in a localized volume is supposed to induce a rise in temperature, which could increase the stacking fault energy and reduce the driving force for the phase transformation [23,27,28,29]. However, the deformation mode of wire drawing is also reported to favor the intersection of mechanical twinning plates and enhance the martensite nucleation, leading to the acceleration of the strain-induced martensitic transformation [21]. Since the mechanism and kinetics of SIMT are sensitive to the steel composition, there is still a lack of specific investigation on the SIMT in the 304 steel grade for wire drawing. This kind of research is critical to the description of the microstructure and to the clarification of strengthening mechanisms of the transformation product.

In this study, a systematic investigation of the microstructure evolution during wire drawing of a 304-type stainless steel was conducted. The kinetics of the strain-induced martensitic transformation has been quantified and analyzed with the Olson–Cohen model. The detailed microstructural characterizations, especially on the nucleation mechanism of α′-martensite, provided a sound basis for an in-depth understanding of the transformation kinetics. In addition, in combination with systematic mechanical testing, the quantitative microstructural characterization also allowed for the delineation of the strengthening contributions of the cold-drawn wire specimens, and the establishment of the structure–property relationship. The outcome of this work provides critical information to formulate guidelines for the optimization of the wire drawing process.

## 2. Materials and Methods

The material investigated in this study was an annealed 304 austenitic stainless steel wire with a nominal composition of Fe–0.02C–18.05Cr–8.41Ni–1.57Mn (wt.%). The initial wire diameter was 100 μm. It was annealed at 900 °C, followed by air cooling. The process line speed during annealing was 30 m/min. Cold wire drawing was performed through a multi-pass process to reduce the wire diameter, with a drawing speed of 100 m/min. The wire drawing process was conducted at room temperature, but a temperature rise could be induced by local deformation at each drawing pass. The equivalent strain was defined as *ε^eq^* = 2ln(*d*_0_/*d_w_*) [3], where *d*_0_ and *d_w_* are the initial and instantaneous wire diameters, respectively. The wire specimens were obtained at different stages of deformation, corresponding to equivalent strains of 0, 0.18, 0.45, 0.71, 1.02, 1.39, 1.83, and 2.41, respectively. The maximum equivalent strain in the present study (2.41) corresponds to a final diameter of 30 μm. The deformation process involved a total of 23 passes of die drawing. The detailed information for the whole wire drawing process is provided in the Supplementary Materials.

Uniaxial tensile tests were conducted at room temperature using an Instron-34TM testing machine (Instron Corporation, Norwood, MA, USA). The specimens had a gauge length of 200 mm and were tested at a constant crosshead speed of 3 mm/min. Three specimens for each microstructure condition were tested. The wire specimens were clamped using gas-operated grippers during tensile testing. The fracture surfaces were examined using secondary electron microscopy (SEM).

Microstructural characterization was performed on the transverse cross-sections of the wires using the Electron backscatter diffraction (EBSD). Specimens were prepared by mechanical polishing followed by electrolytic polishing in 10 vol.% perchloric acid in ethanol at −30 °C under 20 V. EBSD measurements were conducted on a ZEISS Gemini field-emission scanning electron microscope (Carl Zeiss Microscopy GmbH, Oberkochen, Germany) at 20 kV with a step size of 70 nm. The data were processed using AZtecCrystal software (Version 2.1).

For nanoscale microstructural analysis, transmission electron microscopy (TEM) and transmission Kikuchi diffraction (TKD) were employed. Thin films for TEM and TKD were sampled from the wire cross-sections using focused ion beam (FIB) milling. TEM characterization was performed on a Thermo Fisher Talos F200X microscope (Thermo Fisher Scientific Inc., Waltham, MA, USA) at 200 kV. TKD was performed on a Thermo Fisher Scios 2 SEM (Thermo Fisher Scientific Inc., Waltham, MA, USA) at 30 kV with a 5 nm step size.

Phase identification and quantitative analysis were carried out using wide-angle X-ray diffraction (XRD) on a diffractometer equipped with a NANOPIX-WE detector (Rigaku Corporation, Tokyo, Japan), operating at 45 kV with Mo Kα radiation. In this technique, the X-rays are transmitted through the wire specimens, and the complete Debye–Scherrer rings are collected using a two-dimensional detector. As a result, diffraction from a wide range of crystallographic orientations is recorded. By performing azimuthal integration over the entire Debye–Scherrer rings, an orientation-averaged diffraction profile is obtained. Therefore, the influence of texture can be significantly reduced, although not completely eliminated, compared to conventional XRD measurements. The X-ray spot size was 200 μm, which is comparable to the wire diameter, ensuring that the collected signal is representative of the bulk material. Each point corresponds to a single XRD measurement, and the absence of replicates would constitute a limitation of the quantitative analysis. Peaks of (110)_α′_, (200)_α′_, (211)_α′_, (111)_γ_, (200)_γ_, (220)_γ_, and (311)_γ_ were used to calculate the volume fraction of the phases using the following equation [30]:

(1) $$f_{\alpha \prime }=\frac{\frac{1}{n}\sum_{j=1}^{n}\frac{I_{\alpha \prime }^{j}}{R_{\alpha \prime }^{j}}}{\frac{1}{m}\sum_{j=1}^{m}\frac{I_{\gamma }^{j}}{R_{\gamma }^{j}}+\frac{1}{n}\sum_{j=1}^{n}\frac{I_{\alpha \prime }^{j}}{R_{\alpha \prime }^{j}}}$$

where the subscript *j* indicates the *j*-th diffraction peak, *m* and *n* indicate the numbers of diffraction peaks of austenite and martensite, respectively. *R* is the material scattering factor without texture, and *I* indicates the integrated intensities of the diffraction peaks. Note that the uncertainty associated with peak fitting and deconvolution could arise from background subtraction, peak profile selection, and the choice of initial fitting parameters. Instrumental broadening was corrected prior to the analysis.

## 3. Results

**Figure 1:**
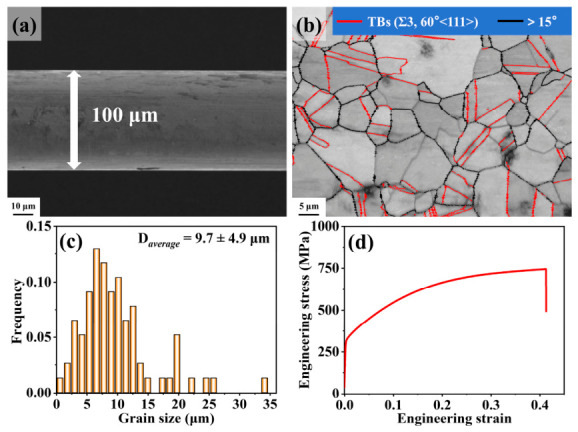
Characterization of the as-received wire. (**a**) SEM micrograph of the surface quality; (**b**) Band contrast map with the distribution of high-angle grain boundaries (HAGBs) and twin boundaries, (**c**) Histogram of austenite grain size distribution; (**d**) Engineering tensile curve.

Figure 1 presents the external and internal structural characteristics of the as-received 304 stainless steel wire. The as-received 304 stainless steel wire has a diameter of 100 μm with a smooth surface, as shown in Figure 1a. The prior annealing produced an equiaxed austenitic microstructure containing annealing twins, with an average grain size of 9.7 μm (Figure 1b,c). Mechanical testing reveals a yield strength of 320 MPa and an elongation to failure of 0.43, indicating high ductility and good drawability (Figure 1d).

**Figure 2:**
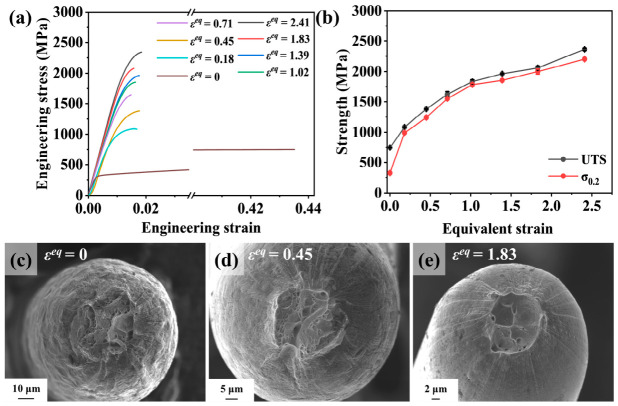
Mechanical properties of the cold-drawn wire specimens. (**a**) Engineering stress–strain curves of wire specimens drawn to different equivalent strains; (**b**) Evolution of yield strength and tensile strength with equivalent strain; (**c**–**e**) Tensile fracture surfaces of the wire specimens at *ε^eq^* = 0, 0.45, and 1.83, respectively.

The mechanical properties of the wire specimens subjected to cold drawing are summarized in Figure 2 and Table 1. The mechanical properties reported are the average values of three tests per condition. Both the yield and tensile strengths increase monotonically with the equivalent strain. At *ε^eq^* = 0.18, the yield strength reaches approximately 1000 MPa, while the elongation is significantly compromised (<0.02). At *ε^eq^* = 1.83, the yield strength already approaches 2 GPa and the ultimate tensile strength is nearly equal to the yield strength, indicating failure immediately after yielding. Although the load drop induced by the occurrence of necking is not obvious in the tensile curves, significant necking with large local deformation is observed in all the wire specimens after tensile testing (Figure 2c–e), including the as-annealed and the severely cold-drawn ones. The fracture surfaces are covered by dimples, indicating a ductile failure mechanism controlled by void nucleation, growth and coalescence.

**Figure 3:**
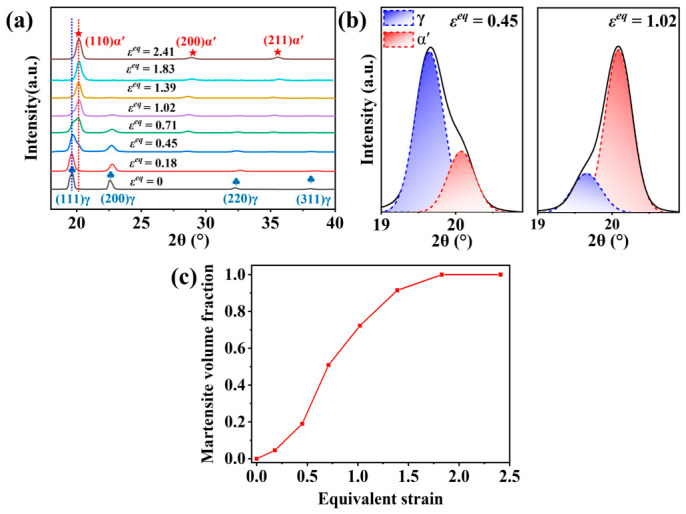
Strain-induced martensitic transformation evaluated by XRD. (**a**) Evolution of XRD patterns with increasing strain, with the location of (111)_γ_ and (110)_α′_ marked by the blue and red dashed lines; (**b**) Representative peak fitting and deconvolution for austenite and α′-martensite phases; (**c**) Evolution of α′-martensite volume fraction as a function of equivalent drawing strain.

The occurrence of strain-induced martensitic transformation is examined using wide-angle X-ray diffraction, which reduces the influence of deformation-induced texture. Diffraction peaks corresponding to austenite ((111)_γ_, (200)_γ_, (220)_γ_, (311)_γ_) and α′-martensite ((110)_α′_, (200)_α′_, (211)_α′_) were analyzed via peak fitting and deconvolution to obtain the phase fractions (Figure 3b). ε-martensite could not be detected by XRD throughout the drawing process. The evolution of the martensite fraction with the equivalent strain displays a typical sigmoidal behavior (Figure 3c). At low strains (<0.75), the transformation proceeds rapidly, whereas at higher strains, the transformation rate decreases progressively. The α′-martensitic transformation is essentially completed at ab equivalent strain of 1.83, which means that the subsequent deformation at a larger strain is carried out by the α’-martensite phase alone.

**Figure 4:**
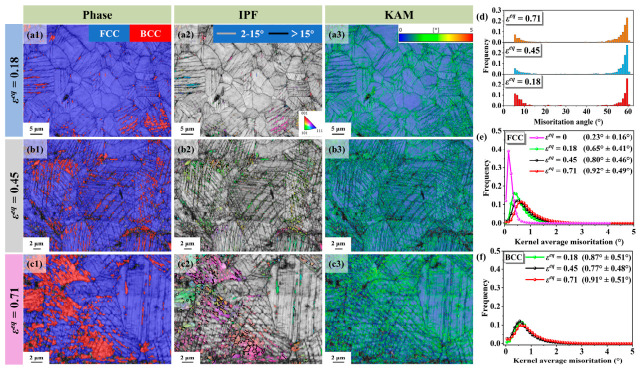
The EBSD characterization of the wire specimens at equivalent drawing strains of 0.18, 0.45 and 0.71, respectively. (**a1**–**a3**) Phase map, IPF of α′-martensite and KAM map at *ε^eq^* = 0.18; (**b1**–**b3**) Phase map, IPF of α′-martensite and KAM map at *ε^eq^* = 0.45; (**c1**–**c3**) Phase map, IPF of α′-martensite and KAM map at *ε^eq^* = 0.71; (**d**) Misorientation angle distributions of α′-martensite; (**e**,**f**) KAM distributions for austenite and α′-martensite, respectively.

**Figure 5:**
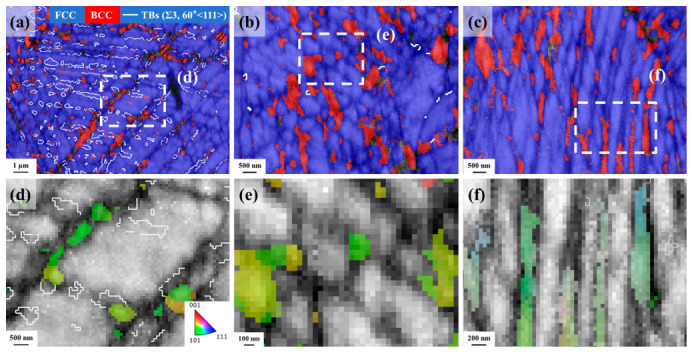
Magnified EBSD maps of the wire specimen at *ε^eq^* = 0.45. (**a**–**c**) Phase maps of selected regions with twin boundaries superimposed; (**d**–**f**) IPF maps of α′-martensite in the selected areas.

The microstructures of the cold-drawn wire specimens were examined to elucidate the mechanisms of the strain-induced martensitic transformation. Figure 4 presents representative microstructures for specimens drawn to equivalent strains of 0.18, 0.45, and 0.71, respectively, corresponding to the progress from the initial transformation stage to a stage dominated by α′-martensite. At an equivalent strain of 0.18 (Figure 4(a1–a3)), significant dislocation accumulation is observed, as indicated by the kernel average misorientation (KAM) distribution, primarily localized at the austenite grain boundaries and within transgranular shear bands. In several austenite grains, the martensitic transformation is initiated at the intersections of these shear bands. With an increased equivalent strain of 0.45 (Figure 4(b1–b3)), the dislocation accumulation continues, and α′-martensite forms throughout all the austenite grains. At *ε^eq^* = 0.71 (Figure 4(c1–c3)), the transformation appears to be dominated by the growth of α′-martensite. In addition to growth along shear bands, impingement between neighboring martensite variants leads to the formation of a blocky morphology. Misorientation analysis (Figure 4d) reveals that martensite variants with the misorientation angles from 5° to 10° and from 55° to 60° are preferably developed at this stage. The plastic deformation of the austenitic matrix is reflected by an increase in KAM values (Figure 4e), whereas no significant increase in KAM is observed in the α’-martensite (Figure 4f). ε-martensite could not be observed in the EBSD results. Figure 5 provides the magnified views of the transformation products for the specimen drawn to *ε^eq^* = 0.45. The activation of multiple shear band systems is highlighted. These shear bands correspond to deformation twins or nanoscale dislocation substructures. The formation of α′-martensite is clearly associated with and confined within these shear bands. Analysis of the inverse pole figure (IPF) maps shows that multiple α′-martensite variants are formed within each shear band system, and the growth of these variants is restricted by their mutual impingement.

**Figure 6:**
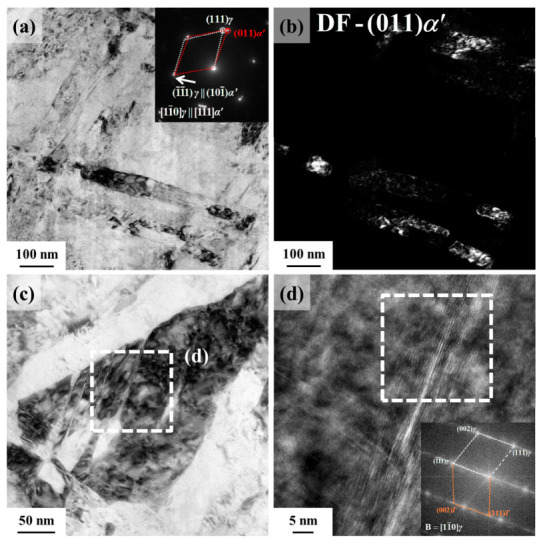
TEM observations of the wire specimen under *ε^eq^* = 0.45. (**a**) Bright-field TEM image showing deformed austenite with shear bands; (**b**) Dark-field TEM image highlighting α′-martensite distribution along shear bands; (**c**) BF-TEM image of austenite; (**d**) HR-TEM image showing the nano-sized twin in austenite.

Figure 6 illustrates the nanoscale microstructural features at an equivalent drawing strain of 0.45. The bright-field TEM (BF-TEM) micrograph (Figure 6a) reveals a deformed austenitic matrix characterized by prominent shear band structures. The corresponding selected area electron diffraction (SAED) pattern (Figure 6a) confirms the classic Kurdjumov–Sachs (K-S) orientation relationship between parent austenite and α′-martensite, matching the parallel orientation relationship of ${\left(\bar{1}\bar{1}1\right)}_{\gamma }\|{\left(10\bar{1}\right)}_{\alpha \prime }$ and ${\left[1\bar{1}0\right]}_{\gamma }\|{\left[1\bar{1}1\right]}_{\alpha \prime }$. The corresponding dark-field TEM (DF-TEM) image (Figure 6b), obtained using the (011)_α′_ diffraction spot, directly visualizes the spatial distribution of α′-martensite, which appears as fine particles within the shear bands and progressively develops into lath-like martensite along the shear band networks. Another localized BF-TEM image captures a residual austenite island. High-resolution TEM (HR-TEM) imaging of the selected region (Figure 6d) indicates that the austenite is heavily faulted, exhibiting the formation of nano-sized twins.

**Figure 7:**
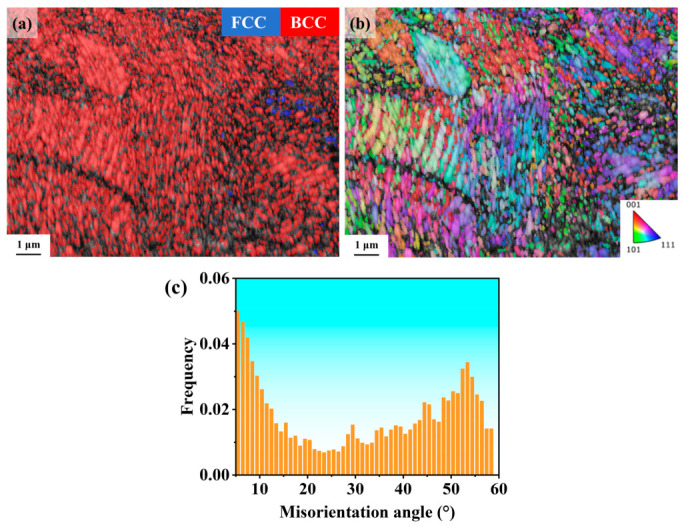
EBSD characterization of the wire specimens at the equivalent strain of 1.02. (**a**) Phase map; (**b**) IPF of α′-martensite; (**c**) Histogram of misorientation angle distribution of α′-martensite.

The microstructure of the wire specimen cold-drawn to an equivalent strain of 1.02 is presented in Figure 7. Despite the large plastic deformation, the quality of the Kikuchi patterns remains sufficient for reliable EBSD analysis. The examined region reveals a microstructure in which the martensitic transformation is nearly completed. A high density of shear bands has effectively subdivided the prior austenite grains, and the subsequent nucleation and growth of α′-martensite along these shear bands produce the characteristic banded morphology. Analysis of the misorientation angle distribution (Figure 7c) indicates that additional martensite variants are significantly formed, in comparison with the earlier-stage microstructures shown in Figure 4d.

**Figure 8:**
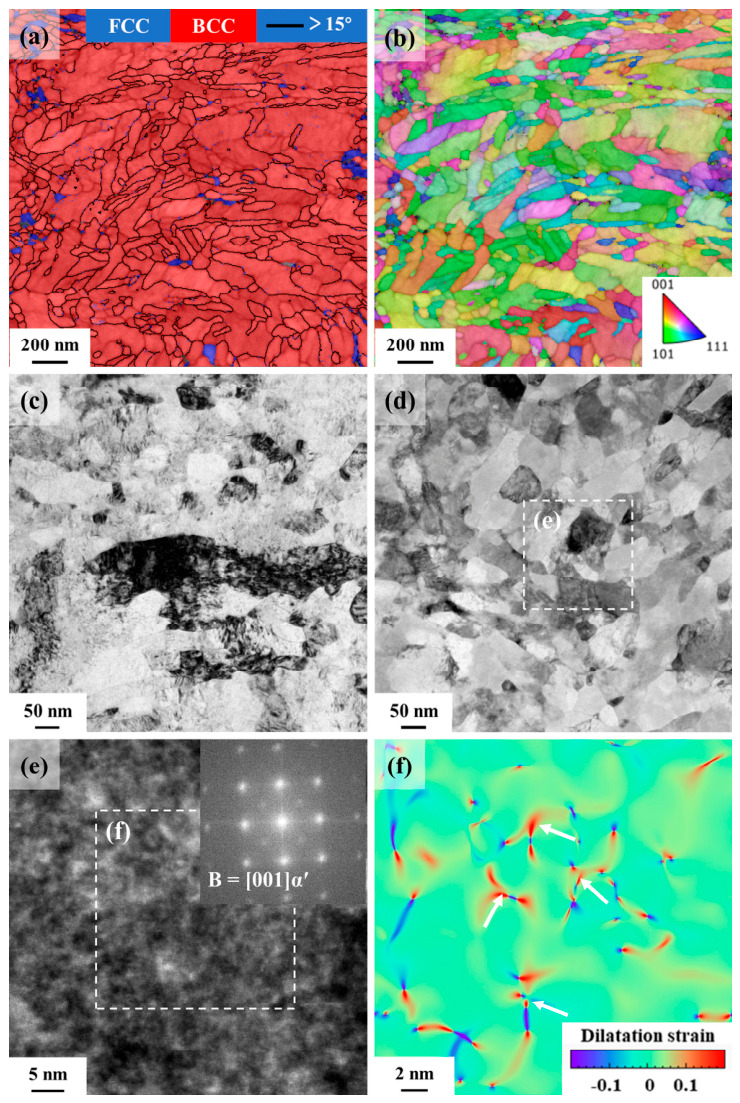
Microstructure of the wire specimen at *ε^eq^* = 1.39. (**a**) TKD phase map showing α′-martensite and residual austenite; (**b**) IPF map of α′-martensite; (**c**,**d**) BF-TEM images showing refined microstructure; (**e**) HRTEM image of selected region; (**f**) GPA dilatation map indicating high dislocation density in α′-martensite, with the dislocations marked by the white arrows.

Figure 8 presents the microstructure of the wire specimen cold drawn to the equivalent strain of 1.39. Due to the nano-scale of the microstructure and the high dislocation density, transmission Kikuchi diffraction (TKD) is employed instead of EBSD. In contrast to the band-type morphology observed at lower strains (Figure 6), the microstructure at this strain exhibits a curled morphology on the transverse cross-section, which is characteristic of wire drawing processes [19,31]. For the α′-martensite, severe plastic deformation has refined the average grain size to 68 nm. At this strain level, martensitic transformation is still incomplete, with the residual austenite islands persisting within the microstructure. The high dislocation density within the α′-martensite is further evidenced by geometric phase analysis (GPA) performed on the high-resolution TEM micrographs.

## 4. Discussion

Starting from the as-annealed state with a grain size of 9.7 μm, the 304 stainless steel exhibits a low yield strength and high ductility, indicating excellent drawability. The relatively low stacking fault energy (~21 mJ/m^2^ of this alloy) favors planar slip, which is indicated by the straight slip lines observed in the EBSD characterization (Figure 4). These slip lines, highlighted in the band contrast maps, are closely associated with microscopic shear bands. The intersections of these shear bands act as the preferential nucleation sites for α′-martensite, as evidenced by the localized formation of martensite at these regions (Figure 4 and Figure 5). With increasing equivalent strain, the density of shear bands progressively increases, leading to a corresponding rise in the number of martensite nucleation sites and, consequently, a continuous increase in the martensite volume fraction. This transformation behavior is generally in agreement with the observations for the 304-type stainless steels in the literature [8,10,32,33,34].

However, distinct differences in the transformation mechanism are identified for wire drawing. In conventional deformation modes, such as cold rolling or uniaxial tension, the martensitic transformation in the 304-type steel grades usually proceeds via the sequence of austenite → ε-martensite → α′-martensite, where the shear bands are primarily composed of faulted ε-martensite [33,34]. In contrast, the ε-martensite was not detected within the resolution and sensitivity limits of the techniques used. This discrepancy can be attributed to the local temperature rise during rapid wire drawing, as also reported in Ref. [23], which increases the stacking fault energy [9]. Such a thermal influence is a plausible hypothesis, although it is challenging to directly measure and verify in this study. Future thermal measurements or thermomechanical simulations would be necessary to confirm this effect. Instead, the shear bands are dominated by deformation twins, and the martensite nucleation is activated at twin intersections, resulting in a modified transformation pathway of austenite → twin → α′-martensite. This change in the transformation sequence highlights the strong influence of the deformation mode on the underlying transformation mechanism.

**Figure 9:**
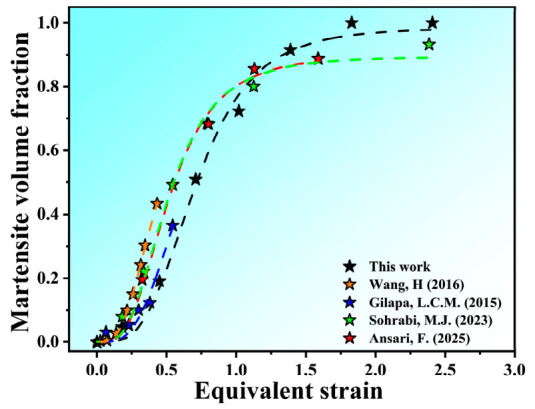
The Olson–Cohen analysis of the data of transformation kinetics during wire drawing. Literature data from Refs. [35,36,37,38] on the plastic processing of 304-type steels were also provided and analyzed.

Based on the above analysis, the martensitic transformation was then quantitatively analyzed using the Olson–Cohen model [15]. In this model, the martensite formation is assumed to occur preferentially at shear band intersections and is expressed as:

(2) $$f_{\alpha^{\prime }}=1-\exp\left\{-\beta {\left\{1-\exp\left(-\alpha \varepsilon \right)\right\}}^{n}\right\}$$

where *f*_α′_, *ε* and *n* are the volume fraction of α′-martensite, the equivalent strain and the exponent, respectively (usually set as 4.5 for the austenitic stainless steels [4,6,15]). The model provides a satisfactory fit to the experimental data over the full strain range, including both the acceleration and saturation stages (Figure 9). Notably, the kinetic parameters identified in the present study differ from those reported for conventional deformation processes, as shown by the data summarized in Table 2. A sensitivity analysis is provided in the Supplementary Materials, which suggests that the parameters *α* and *β* are only weakly influenced by variations in the value of *n*. The postponement of the transformation to larger strains, as shown in Figure 9, results in a reduced α value, while the sustained formation to nearly completion suggests a higher *β* value. The reduced *α* value reflects a lower rate of shear band formation, presumably as a result of the rise in temperature. But the elevated *β* value indicates an increased probability of martensite nucleation at shear band intersections. This behavior is substantiated by the enhanced formation of intersections of twin-related shear bands formed during wire drawing. This is consistent with the reported trend comparing the SIMT in 316 during cold rolling and cold wire drawing [21].

The evolution of the mechanical properties is closely correlated with the underlying microstructural changes. A continuous increase in the yield strength is observed with increasing equivalent strain, reaching approximately 1 GPa at *ε^eq^* = 0.18 and 2.0 GPa at *ε^eq^* = 1.83, respectively. This pronounced strain hardening originates from both dislocation accumulation and phase transformation, although their contributions are not equivalent. The evolution of KAM values suggests that dislocations accumulate in the austenite phase during deformation, contributing to strengthening at low to intermediate strains. However, the rate of KAM increase becomes less significant beyond *ε^eq^* = 0.45, indicating a gradual saturation of dislocation storage. In contrast, the martensite phase exhibits a negligible KAM evolution, which could be attributed to its intrinsically high dislocation density and a limited capacity for further accumulation [39].

**Figure 10:**
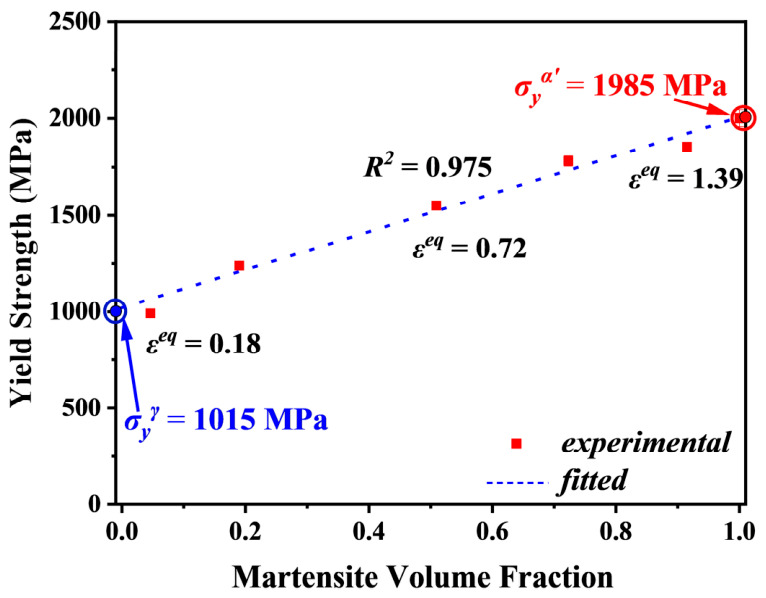
Correlation analysis of yield strength with martensite volume fraction. The phase strengths could be estimated when extending to the situations of fully austenite and fully α’-martensite.

More importantly, a linear relationship between the yield strength and the martensite volume fraction is observed (Figure 10), with a high regression coefficient of 0.975. In this sense, the yield strength of the cold-drawn wire specimen could be approximately described by the following rule-of-mixture equation:

(3) $$\sigma_{0.2}=f_{\alpha^{\prime }}\sigma_{y}^{\alpha^{\prime }}+\left(1-f_{\alpha^{\prime }}\right)\sigma_{y}^{\gamma }$$

where $\sigma_{y}^{\alpha^{\prime }}$ and $\sigma_{y}^{\gamma }$ are the yield strengths of martensite and austenite, respectively, and $f_{\alpha^{\prime }}$ is the martensite volume fraction. This relationship indicates that the strengthening behavior is, as a first-order approximation, governed by a composite effect [40,41]. According to the rule of mixtures, the yield strength of the material can be expressed as the weighted contribution of martensite and austenite phases. The inversely estimated phase strengths, 1985 MPa for α′-martensite and 1015 MPa for austenite, reveal a significant strength contrast, which leads to pronounced stress partitioning between the two phases. The elevated strength of the austenite compared to the as-annealed state is attributed to prior plastic deformation before the martensitic transformation. The resulting microstructure after partial transformation is thus essentially composed of a mixture of α’-martensite and heavily deformed austenite. The estimated strength level of α′-martensite is higher than the strength level of the martensite phase (1.4 GPa) obtained by TRIP in the 301LN steel grade [42] and that (1.6 GPa) in 316L [40], and it approaches the magnitude in iron (2 GPa) subjected to severe plastic deformation up to an equivalent strain of 10.0 [43,44]. Usually, α′-martensite without significant interstitial content exhibits moderate strength [45,46]. In the present case, however, α′-martensite is formed from a highly deformed austenite matrix with a high pre-existing dislocation density. These dislocations are likely inherited during the phase transformation, providing an additional strengthening contribution to the martensite phase [47,48]. This inheritance effect, combined with the dynamic composite strengthening, accounts for the exceptional strength achieved in the wire-drawn 304 stainless steel. Note that these values do not represent a direct determination of the intrinsic strength of each phase, but rather an effective estimate obtained from a macroscopic fitting. The actual strength may be influenced, as discussed above, by the dislocation density, grain size, texture, internal stresses and load partitioning between the phases.

## 5. Conclusions

This study has clarified the microstructural evolution, strain-induced martensitic transformation and strengthening mechanisms of 304 stainless steel under cold wire drawing. The α′-martensite is formed preferentially at shear band and twin intersections, with multiple variants developing along the shear bands. The formation of ε-martensite is negligible, as it cannot be detected within the resolution and sensitivity limits of the techniques used. The austenite transforms via a twin-assisted pathway (austenite → twin → α′-martensite). The kinetics of the transformation during wire drawing were quantified by the XRD method and follow the Olson–Cohen model. The yield strength of the cold-drawn wire specimens increases linearly with the martensite fraction, and is governed by a composite effect in the mixture of α′-martensite (estimated strength of 1985 MPa) and the highly deformed austenite (estimated strength of 1015 MPa). It is worth further exploring the effects of alloy composition and initial microstructure on the kinetics of SIMT and the mechanical properties, which will enable optimization in the control of die wear and the in-use properties of stainless steel wires.

## Figures and Tables

**Table 1:** Mechanical properties of the cold-drawn wire specimens.

Equivalent Strain	σ_0.2_	UTS	Equivalent Strain	σ_0.2_	UTS
0	327 ± 2	747 ± 2	1.02	1782 ± 28	1835 ± 31
0.18	991 ± 20	1085 ± 1	1.39	1855 ± 15	1963 ± 2
0.45	1239 ± 22	1378 ± 3	1.83	2000 ± 42	2061 ± 29
0.71	1551 ± 14	1622 ± 46	2.41	2202 ± 2	2359 ± 18

**Table 2:** Summary of parameters of Olson–Cohen analysis of different materials.

Composition	Deformation Mode	Measurement Technique	*α*	*β*	R^2^	References
Fe–0.02C–18.05Cr–8.41Ni–1.57Mn	Drawing	XRD	1.53	4.27	0.995	This work
Fe–0.08C–19Cr–9.25Ni–2Mn	Tensile	Neutron diffraction	3.45	1.79	0.999	[35]
Fe–0.063C–18.27Cr–8.1Ni–1.007Mn	Tensile	Magnetic measurement	1.97	2.92	0.946	[36]
Fe–0.01C–18.6Cr–8.3Ni–1.4Mn	Rolling	XRD	2.68	2.22	0.992	[37]
Fe–0.046C–17.91Cr–9.16Ni–1.54Mn	Rolling	XRD	2.62	2.26	0.996	[38]

## Data Availability

The original contributions presented in this study are included in the article and Supplementary Material. Further inquiries can be directed to the corresponding authors.

## Supplementary Materials

The following supporting information can be downloaded at: https://www.mdpi.com/article/10.3390/ma19112412/s1, Table S1: List of parameters of the wire drawing process; Figure S1: Sensitivity analysis of the exponent n in the Olson–Cohen model; Table S2: Fitting parameters *α*, *β*, and *R*^2^ obtained with different values of *n*.
