# Supplementary material for: Martensitic Transformation and Strengthening Mechanism in a 304 Stainless Steel Subjected to Wire Drawing
Source: Materials (Basel). 2026 Jun 5;19(11):2412. doi: 10.3390/ma19112412 (PMC13258671; doi:10.3390/ma19112412)
Supplement: Supplementary file 1 [file materials-19-02412-s001.zip › materials-4331077-supplementary.pdf]

Supplementary Materials

Martensitic transformation and strengthening mechanism in a 304 stainless steel subjected to wire drawing

Yongjie Yu[1], Wujing Fu[1], Feng Dai[1], Rengeng Li[1,*], Qingquan Lai[1,*]
[1]Key Laboratory for Light-Weight Materials, Nanjing Tech University, Nanjing, China
*Corresponding author, E-mail: qingquanlai@hotmail.com (Q. L.); lirengeng@njtech.edu.cn (R.L.)

1. Table S1 provides the detailed parameters, including the wire diameter, the reduction in the sectional area per pass and the accumulated equivalent strain for the whole wire drawing process. The drawing die features a single-crystal diamond inner core, with an approach angle of 10–13°and an exit angle of 60° (both defined as half-angles relative to the die axis). A water-based lubricant is used in an immersion mode to cool and lubricate the die orifice during drawing.

Table S1 List of parameters of the wire drawing process

Pass	Diameter (μm)	Reduction per pass	Accumulated equivalent strain	Pass	Diameter (μm)	Reduction per pass	Accumulated equivalent strain
1	97.59	0.05	0.05	13	50.00	0.10	1.39
2	91.20	0.13	0.18	14	47.70	0.09	1.48
3	85.23	0.13	0.32	15	45.52	0.09	1.57
4	80.00	0.12	0.45	16	43.43	0.09	1.67
5	75.95	0.10	0.55	17	40.00	0.15	1.83
6	70.00	0.15	0.71	18	37.74	0.11	1.95
7	66.40	0.10	0.82	19	36.01	0.09	2.04
8	63.10	0.10	0.92	20	34.37	0.09	2.14
9	60.00	0.10	1.02	21	32.79	0.09	2.23
10	58.51	0.05	1.07	22	31.29	0.09	2.32
11	55.53	0.10	1.18	23	30.00	0.08	2.41
12	52.71	0.10	1.28				

2. A sensitivity analysis has been conducted on the effect of the value of n on the fitting of transformation kinetics and the identification of α and β values. It is shown in Figure S1 that a satisfactory fitting by the Olson–Cohen model can be obtained with the values of n from 4.0 to 5.0. The fitting parameters are listed in Table S2.

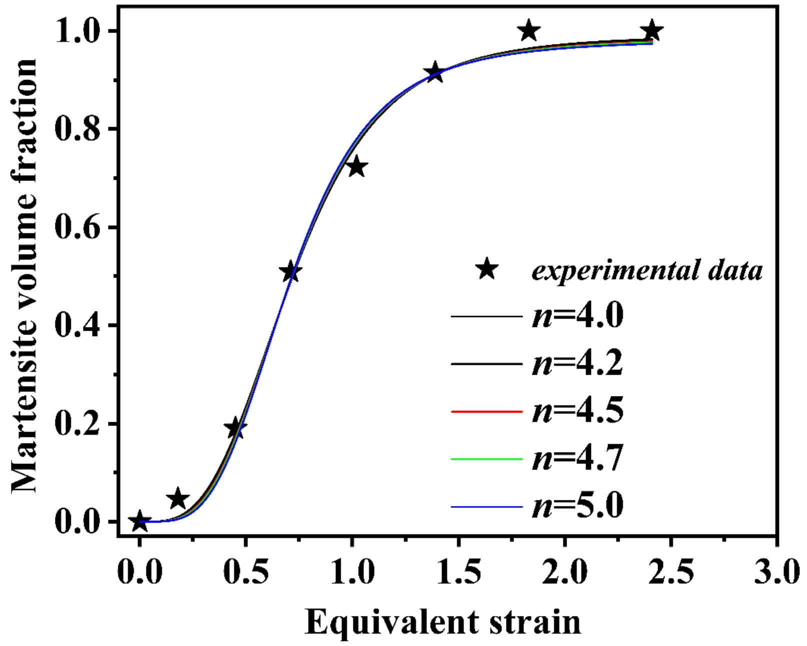

Figure S1 Sensitivity analysis of the exponent n in the Olson–Cohen model

Table S2 Fitting parameters α, β, and R^2 obtained with different values of n

n	α	β	R^2
4.0	1.33	4.80	0.996
4.2	1.42	4.55	0.995
4.5	1.53	4.27	0.995
4.7	1.61	4.12	0.994
5.0	1.71	3.94	0.992
